# Exploring the Motivational Roots of Getting Vaccinated against COVID-19 in a Population of Vaccinated Pediatric Healthcare Professionals: Evidence from an Italian Cross-Sectional Study

**DOI:** 10.3390/vaccines10030467

**Published:** 2022-03-18

**Authors:** Serena Barello, Giuseppe Maiorino, Lorenzo Palamenghi, Chiara Torri, Marta Acampora, Luigi Gagliardi, SPACE NET Study Group

**Affiliations:** 1 EngageMinds HUB–Consumer, Food & Health Engagement Research Center, Università Cattolica del Sacro Cuore, 20123 Milan, Italy; lorenzo.palamenghi@unicatt.it (L.P.); chiara.torri02@icatt.it (C.T.); marta.acampora01@icatt.it (M.A.); 2 Department of Psychology, Università Cattolica del Sacro Cuore, 20123 Milan, Italy; 3 Division of Neonatology and Pediatrics, Ospedale Versilia, 54027 Viareggio, Italy; giuseppe15maiorino12@gmail.com (G.M.); luigi.gagliardi@uslnordovest.toscana.it (L.G.); 4 Faculty of Agriculture, Food and Environmental Sciences, Università Cattolica del Sacro Cuore, Via Milano 24, 26100 Cremona, Italy

**Keywords:** vaccine hesitancy, pediatric healthcare workers, COVID-19, vaccine acceptance, applied psychology, healthcare professionals

## Abstract

Health care professionals (HCPs) working in pediatric and perinatal settings have a strong influence on parental vaccine decision making. Furthermore, HCPs’ motivations behind vaccine acceptance are associated with their likelihood of recommending vaccines to their patients. Understanding these motivations in the context of the COVID-19 vaccination campaign may aid in the development of interventions that improve pediatric practitioners’ vaccine confidence and prescription. We aimed at studying the motivations affecting COVID-19 vaccination behavior among a sample of vaccinated Italian HCPs working in pediatric settings. A sample (*n* = 162) of HCPs completed an online self-reported survey exploring motivations behind getting vaccinated against COVID-19. Emotions of HCPs at the moment of COVID-19 vaccination injection were also recorded to collect data about the main feelings connected to the vaccination decision-making process. Data were collected between 19 March 2021 and 21 April 2021. The most effective motivational incentives were the beliefs that vaccination helped protect vulnerable members of the community (97.5% agreement), could protect one’s own health (93.7%), health authorities could be trusted (58.7%), and the vaccine had been rigorously tested (53.8%). Actual personal exposure to COVID was less important (reported importance agreement 16–24%), and the influence of news and social media was still lower (4–6%). Differences between physicians’ and other HCPs’ ratings were also found. Finally, emotional status at vaccination showed high ratings for positive emotions surrounding the vaccination act. This study provided additional evidence about the multifaceted motivations behind COVID-19 vaccine acceptance and showed the potential of understanding the psychosocial roots of vaccine behaviors for shaping public communication campaigns. The highly emotionally charged response obtained underscores the importance of strengthening the community feeling among HCPs.

## 1. Introduction

The COVID-19 vaccine is considered critical to control the pandemic. At the time of writing this manuscript (i.e., March 2022), more than five million deaths have been recorded since the beginning of the COVID-19 pandemic, with millions more infected and many with related morbidities [1]. In this scenario, massive vaccination against this disease has significantly decreased the burden of the pandemic. Its role in the COVID-19 spread has been widely recognized, and a high vaccination rate is needed to bring the pandemic under control. Many studies across countries have been conducted to understand peoples’ willingness to take the COVID-19 vaccine. Globally, a declining trend of COVID-19 vaccination intent has been reported [2]. In particular, several studies discovered a number of different factors as drivers of vaccination behaviors [3,4,5,6]. Major public concerns relate to the safety and efficacy of the vaccines and fear of possible side effects. Additionally, lack of trust in healthcare agencies and in the vaccine’s efficacy has been identified as a determinant of vaccine hesitancy [7].

Besides these general results, authors observed high heterogeneity in responses among countries and populations, highlighting the need to explore such variation and to understand and cope with group-specific concerns [8].

However, regardless of the well-renowned importance of promoting COVID-19 vaccination, vaccine hesitancy has increasingly become a global concern and a crucial factor in under-vaccination [9]. Despite the global effort to end the COVID-19 pandemic, anti-vaccination sentiments, fostered by the spread of misinformation on the dangers and consequences of vaccination, may cause hesitancy in immunization against preventable infectious diseases [10]. Vaccine hesitancy is a common phenomenon in Western countries, with Italy having one of the highest rates of non-compliance in regards to vaccination campaigns in Europe [11,12].

In this challenging scenario, healthcare professionals (HCPs) constitute one of the primary targets for vaccination promotion and advocacy from the perspective of public health agencies. The World Health Organization (WHO) has prioritized HCPs receiving the COVID-19 vaccine, particularly in cases of limited vaccine dose availability [13,14]. They are one of the population groups most likely to be infected and they can thus spread the contagion to other people [15]. Some studies revealed that HCPs are three times more at risk of being infected with COVID-19 than the general population [16]. Promoting vaccination intention in HCPs is thus vital for greater public vaccination acceptance, as patients demonstrate high trust in vaccinators [17]. On the contrary, HCPs’ hesitancy may be a large barrier for the effectiveness of the massive COVID-19 vaccination campaign [18,19].

Moreover, HCPs are expected to be aware of the vaccination-related risks, to be able to weigh them against the risks of contracting preventable diseases, and to communicate this topic to patients and families they take care of in the most effective way [20]. Other studies have demonstrated that there is a relationship between the attitudes and feelings of HCPs toward vaccines and their vaccine recommendation behavior to patients [21,22,23]. The literature also reports that HCPs can themselves be vaccine-hesitant [24], and that their hesitancy can impact hesitancy among the general public as well [25,26,27]. Furthermore, other studies pointed out the role of emotional engagement in shaping vaccination attitudes and behaviors [28]. Indeed, even before the COVID-19 pandemic, vaccination had long been an emotionally charged issue in many lay and professional people. Thus, efforts toward coping with vaccine hesitancy and increasing trust in vaccination behaviors need to include attention to the emotional response evoked by this preventive requirement [29,30,31]. 

In pediatric settings, HCPs are consistently mentioned as a key factor in parental vaccine decision making and as a trusted source of vaccine information [32,33,34], even among parents who are unsure about vaccines [32]. Thus, given the crucial role that pediatric practitioners’ attitude toward vaccination has on shaping parental decision making on vaccines, this study aims to understand the main motivations behind pediatric/perinatal HCPs’ COVID-19-related vaccination behaviors in a sample of Italian vaccinated practitioners. Moreover, the study also investigated the emotions related to the act of vaccination as they can offer insights about the role of feelings in the vaccine-related decision-making process. It is of utmost importance to investigate motivational factors and incentives that affect HCPs’ intention to get the COVID-19 vaccine, especially those psychosocial and emotional factors that can be modified through educational and communication interventions.

## 2. Materials and Methods

### 2.1. Study Population and Design 

This study is part of the Staff and Parental Adjustment to COVID-19 Epidemics–Neonatal Experience in Tuscany (SPACE-NET) study, whose primary aims were to investigate the impact of COVID-related stress on HCPs’ wellbeing and HCPs’ response to various aspects of vaccine hesitancy. The study is described in greater detail elsewhere [35].

A sample (*n* = 162) of pediatric HCPs was asked to complete an online self-reported survey. The sample was recruited from the local healthcare services in the Tuscany region (AUSL Toscana Nord Ovest) using a mailing list, and was composed of physicians, nurses, midwives, and allied healthcare professionals working in perinatal and pediatric healthcare services. The online survey focused on the assessment of COVID-19 vaccine hesitancy and uptake, and the identification of the motivations behind acceptance of vaccination among healthcare workers. The survey was delivered by e-mail, which contained an explanation of the research and a link to the platform (Qualtrics) where participants could complete the questionnaire. Data were collected from 19 March 2021 to 21 April 2021, when Italy was facing the last stage of the third wave of COVID-19 infection, and three months after the start of the vaccination campaign for HCPs (27 December 2020), a time when HCPs’ vaccination was not yet mandatory. 

### 2.2. Measures

The survey involved a series of questions about sociodemographic characteristics, whether the participant had received a vaccination against influenza in the preceding year (season 2019–2020), and participants’ actual adherence to the COVID-19 vaccination campaign.

Participants who declared they had been vaccinated or reported the intention to get vaccinated were also asked to answer 11 additional items regarding the reasons that could have motivated them to adhere to the vaccination campaign. In particular, 11 of these items were selected from a previous study [36] carried out to identify the most promising incentives for improving the likelihood of vaccination uptake when a vaccine against COVID-19 would have been available. In our research, we used the list in a post hoc way, in order to identify the incentives that had played a crucial role in the decision to get vaccinated against COVID-19. We added the following question (“*I am convinced that the vaccine will serve to protect my health*”). Moreover, participants were asked to indicate how much they felt in some specific emotional states, when they had received the COVID-19 vaccine, using the scale from 0 (not at all) to 100 (very much).

The whole questionnaire is available in the Supplementary Materials File S1.

### 2.3. Statistics Analyses 

Descriptive statistics were computed: in particular, to rank the effectiveness of the incentives, the percentage of respondents who answered 4 or 5 (yes or definitely yes) was calculated for each item. We then assessed whether there were differences between professional groups in the percentage of people who deemed an incentive important. Thus, participants were divided according to their professional profile (physicians vs. nurses, midwives, and other allied healthcare workers) and contingency tables and Pearson’s chi-square statistics were used with two-tailed significance tests. Results were considered significant with *p* < 0.05.

The same approach was used to assess whether there were differences in motivation between participants who were vaccinated against influenza in the past and participants who did not get vaccinated.

## 3. Results

### 3.1. Sample Characteristics and COVID-19 Vaccine Hesitancy and Uptake 

Participants who were vaccinated or who declared the intention to be vaccinated were the large majority of the sample: 98.8%. Of these, 95.7% had already been vaccinated with the first and second dose; 2.5% were vaccinated only with the first dose. The remaining ones were not vaccinated but they declared the intention to be vaccinated against COVID-19 in the near future. No one reported to be against COVID-19 vaccination. 

Of the 160 HCPs studied, 87.5% were female. Mean age was 45 (range 26 to 65), with a standard deviation (SD) of 10.2. Regarding the occupational profile of the respondents, the majority were physicians (35.6%) or nurses (35.6%), followed by midwives (24.4%), allied HCP (3.1%), and others (1.3%). 

### 3.2. Motivation behind COVID-19 Vaccination Behaviors 

Table 1 shows the percentage of respondents who answered “yes” or “definitely yes” for each item. The most effective motivational incentive was “being convinced that getting vaccinated helped protect vulnerable members of my community”, with 97.5% (*n* = 156) of the sample who agreed or strongly agreed with the statement, followed by “being convinced that the vaccine will serve to protect my health”. An intermediate agreement was obtained by “thinking that the health authorities were trustworthy on this argument”, and “being convinced that the vaccine had been rigorously tested”. The least effective motivational incentives concerned the promotion of vaccine uptake from the President of the Republic or the Prime Minister, from social media, and from the news media.

Table 2 reports the comparison between physicians and other HCPs related to their motivations to get vaccinated. While for many items physicians and other HCPs did not show differences, physicians showed greater trust in the health authorities (70.2% vs. 52.4% among other HCPs, *p* = 0.029), while direct COVID-19 experience (personally knowing people infected, hospitalized, or who died because of COVID-19) was more important among other HCPs than among physicians (see Table 2). Importantly, there were no significant differences between the two groups in terms of actual personal exposure to COVID-19: in the whole sample, 6.9% reported being personally diagnosed with COVID-19 (5.3% for physicians and 7.8% for other professionals, *p* = 0.63), 76.9% reported having personally assisted a patient with COVID-19 (84.2% for physicians and 72.8% for other professionals, *p* = 0.10), and 16.3% reported having lost a relative or a friend due to COVID-19 (17.5% and 15.5% for physicians and other professionals, respectively, *p* = 0.74).

### 3.3. Motivation behind COVID-19 Vaccination Behaviors and Influenza Vaccination Acceptance in the Past 

In sharp contrast with the acceptance of the COVID-19 vaccine, only 51.2% of the sample had been vaccinated against influenza in the past, and only 68.8% in the 2020–2021 season: in particular, in the 2020–2021 season, physicians were vaccinated more frequently than the other HCPs (86% and 59.2%, respectively, *p* < 0.001). No differences by age or gender were found.

The comparison of motivations between those vaccinated and those not vaccinated against influenza in the past is shown in Table 3. 

There was a clear difference between groups only for the incentive “*Being convinced that the vaccine will serve to protect my health*”: the respondents who answered yes/definitely yes were 97.6% among those who were vaccinated against influenza in the past versus 89.7% among those never vaccinated (*p* = 0.041). Trust in healthcare authorities was slightly higher in those vaccinated against the flu (65.9%) than in those non-vaccinated (51.3%), though this did not achieve statistical significance.

**Figure 1:**
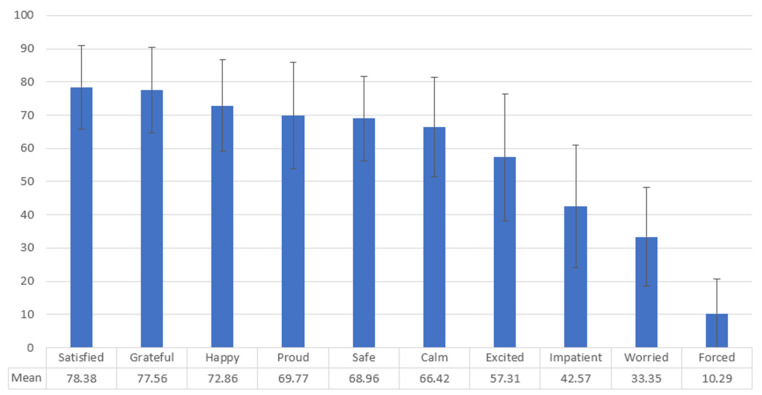
Emotional status (mean) at the moment of vaccination reported by participants (*n* = 159). Bars show standard deviation.

Among those vaccinated against COVID-19, the emotional state after vaccination is reported in Figure 1.

Although a minority of vaccine recipients reported feelings of being forced or worried, a large majority reported strong positive feelings including satisfaction, happiness, and feeling safe, but also feeling grateful and proud.

## 4. Discussion

The present study investigated the main motivational roots behind COVID-19 vaccination behaviors among HCPs working in pediatric settings. Results from this study showed that the most effective motivational incentives were the beliefs that vaccination helped protect vulnerable members of the community and helped protect one’s own health. Moreover, the level of trust in health authorities impacts the HCPs’ decision to get vaccinated. Differences between physicians’ and other HCPs’ ratings were also found. Finally, emotional status at vaccination showed high ratings for positive emotions surrounding the vaccination act. 

The novelty in this study stems from being the first to assess the psychosocial motivations for getting vaccinated against COVID-19 among vaccinated pediatric HCPs working in Italy. The focus on HCPs’ beliefs, attitudes, and emotions surrounding COVID-19 vaccination in this specific clinical setting is relevant for three main reasons: first, HCPs represent a group with a higher risk of contagion. Second, HCPs can play a central role in addressing COVID-19 vaccine hesitancy by educating families on the importance of vaccination in the fight against the ongoing pandemic and other infectious diseases [18]. A third reason is that, while we are writing this manuscript, vaccination has been approved for children older than 12 years, and approval for the 5–11 years span is forthcoming. This is another contextual element that makes healthcare professionals in this field strong influencers on parental vaccine decision making, even (perhaps especially) for those parents and/or HCPs who believe that vaccines are not safe. The results of our study showed that motivations and emotions influencing the COVID-19 vaccination behavior are multiple and refer to different psychosocial levers. In particular, collective responsibility, protecting ones’ own health, and confidence in vaccine effectiveness were associated with a stronger intention to be vaccinated against COVID-19, confirming studies conducted in other HCP populations [37].

The first motivation endorsed by study participants behind their vaccination behavior is related to their sense of collective responsibility since the vaccine is the best way to help protect vulnerable members of their community. 

This is in line with Betsch and colleagues [38] who argue that a sense of collective responsibility and a sense of community may increase one’s willingness to protect others by means of getting vaccinated.

Interestingly, previous vaccination against the flu was associated with a higher rating of the reason “Being convinced that the vaccine will serve to protect my health”. This result, obtained in a sample of HCPs vaccinated against COVID-19, complements the findings that individual perceived risk and flu vaccination during previous seasons were associated with hypothetical COVID-19 vaccine acceptance [39]. 

If we consider those vaccinated the previous year against influenza as “compliers” with recommendations, we expect to witness high compliance with the COVID-19 vaccination as well. Those who were not vaccinated against flu the previous year, however, represent a “new” basin of compliers. However, even in the season 2020–2021, only 68.5% were vaccinated against flu (+17% compared to the previous years), that is, they only modestly changed their opinion on the risk of flu, and of the value of flu vaccination. It is possible that these HCPs accepted vaccination to act as role models for the community. It is not surprising, then, to see that a lower percentage of HCPs, among the non-flu-vaccinated, reported self-protection as a reason for COVID-19 vaccination. 

Taken together, these data indicate that the exceptional situation in which HCPs found themselves during the COVID pandemic has stimulated increased evaluation of risks and priorities, different from the “standard” attitude towards vaccinations. 

The exceptionality of decision making and attitudes in the COVID situation is also witnessed by the highly emotional response toward the vaccination, where recipients have described themselves as “grateful” and “proud”, “excited” and “impatient”. This unusual sentiment in the case of vaccines has been recently highlighted in an editorial in the *NEJM*, titled “in gratitude of mRNA vaccines” [40], but the pride felt by recipients of the vaccine is probably linked with the idea of doing something worthwhile for the community, as reported in many studies on this topic [41,42]. Self-perceived risk of COVID-19 and perceived impact on health were also important motivations.

According to these data and in line with other studies [29,43], acknowledging fears, anger, and other negative emotions and fostering the positive ones while emphasizing the safety and efficacy standards of the COVID-19 vaccine development process may help to increase vaccine confidence.

In alignment with other studies [44,45], a common concern reported by HCPs is that the risks related to getting vaccinated might outweigh the benefits. First, it is interesting to note that despite the uncertainty of the COVID-19 vaccine efficacy in the long term, side effects, and duration of protection at the time of data collection, study participants who were vaccinated and/or declared the intention to be vaccinated were 98.8% of the sample.

This study found that HCPs would be more likely to get vaccinated if they were persuaded that the vaccine had been rigorously tested. To maximize vaccine uptake, health agencies should reassure the public that vaccine development has followed all the preestablished guidelines and that the process of developing a vaccine has not been rushed. Respondents reported in a minority of cases a feeling of being worried or forced. We should reiterate that this study was carried out in March–April 2021, when data on the safety of these vaccines were much scantier.

In addition, the level of trust in authorities seems to be one of the stronger motivations behind COVID-19 vaccine acceptance, confirming previous reports [46], while general and COVID-19-vaccine-specific mistrust in the pharmaceutical industry, authorities, and health policies was also associated with COVID-19 vaccine hesitancy [20]. 

The study results showed that in this sample of HCPs, promotion of vaccine uptake from the social network, trusted news media, or from community/political leaders was less important. This result suggests that, at least in the perspective of this study sample, the contents of the communication could be more relevant compared to the source of the communication when incentivizing the vaccination behavior. This is in line with a previous study conducted in another population [36].

Some limitations of this study should be noted. First, the generalizability of the results must be taken with some caution, as the study is based on a self-selected purposive sample of individuals from a single geographical area, with a mixed attitude toward influenza vaccinations but a strong positive attitude toward COVID vaccines. 

Furthermore, because the results of this research are cross-sectional, longitudinal monitoring of intention to get vaccinated against COVID-19 in the long term (i.e., further doses among the pediatric HCPs population, as well as discipline-specific subgroups (doctors, nurses, midwives, allied HCPs), will be needed as the pandemic rapidly evolves. Moreover, while this study showed the role of some well-known motivations behind the COVID-19 vaccination intentions, it may have lacked in identifying additional factors that may be specific for this particular population. A qualitative study may be useful to broaden our understanding and inform future communication campaigns. Finally, in this study there are no data included on non-vaccinated HCPs, making it difficult to understand the barriers HCPs face when they do not support vaccination, but still need to advise parents. Arguably, this is a relevant research space for future studies on this topic. 

## 5. Conclusions

This study provided additional evidence about the motivations behind COVID-19 vaccine acceptance and showed the potential in understanding the psychosocial roots of vaccine behaviors for shaping public communication campaigns having this target in mind. The lessons learned among Italian pediatric HCPs would be helpful for the development of vaccine campaigns in the rest of the world, especially for other healthcare workers, to prevent or prepare for the next wave of the outbreak or future pandemics and in general to promote vaccination behaviors in this peculiar clinical context. These study results are expected to provide suggestions for projected vaccine uptake and underlying drivers of vaccine-related decision making among HCPs. By greater understanding of this issue, effective strategies can be implemented to foster COVID-19 vaccine uptake in Italy, as well as in other settings. 

## Figures and Tables

**Table 1:** Ranking of the motivations behind getting the COVID-19 vaccine (total analyzed *n* = 160).

Motivations	*n* (%) of Yes/Definitely Yes Answers
Being convinced that getting vaccinated helped protect vulnerable members of my community	156 (97.5%)
Being convinced that the vaccine will serve to protect my health	150 (93.7%)
Thinking that the health authorities were trustworthy on this argument	94 (58.7%)
Being convinced that the vaccine had been rigorously tested	86 (53.8%)
The fact that a trusted health care worker suggested I get vaccinated	45 (28.2%)
The fact that someone I knew got sick with COVID-19	38 (23.8%)
The fact that someone I knew was hospitalized due to COVID-19	35 (21.9%)
The fact that someone I knew died due to COVID-19	25 (15.6%)
The fact that the President of the Republic or the Prime Minister promoted the vaccine	12 (7.5%)
The fact that the vaccination was promoted in my social media network	7 (4.4%)
The fact that a trusted news source promoted the vaccine	9 (5.7%)

**Table 2:** Motivation ranking behind COVID-19 vaccination behavior in physicians and other HCPs (*n* = 160).

Motivation	Physicians (*n*)% Yes/Definitely Yes	Other HCPs (*n*)% Yes/Definitely Yes	Δ%	χ^2^ _(d.f.)_	*p*	Cramer’s V
Being convinced that getting vaccinated helped protect vulnerable members of my community	100% (*n* = 57)	96.1% (*n* = 99)	3.9%	2.270 _(1)_	*p* = 0.132	
Being convinced that the vaccine will serve to protect my health	94.7% (*n* = 54)	93.2% (*n* = 96)	1.5%	0.147 _(1)_	*p* = 0.701	
Thinking that the health authorities were trustworthy on this argument	70.2% (*n* = 40)	52.4% (*n* = 54)	17.8%	4.769 _(1)_	*p* = 0.029	0.173
Being convinced that the vaccine had been rigorously tested	61.4% (*n* = 35)	49.5% (*n* = 51)	11.9%	2.086 _(1)_	*p* = 0.149	
The fact that a trusted health care worker suggested I get vaccinated	28.1% (*n* = 16)	28.2% (*n* = 29)	−0.1%	<0.001 _(1)_	*p* = 0.991	
The fact that someone I knew got sick with COVID-19	14% (*n* = 8)	29.1% (*n* = 30)	−15.1%	4.615 _(1)_	*p* = 0.032	0.17
The fact that someone I knew was hospitalized due to COVID-19	12.3% (*n* = 7)	27.2% (*n* = 28)	−14.9%	4.769 _(1)_	*p* = 0.029	0.17
The fact that someone I knew died due to COVID-19	7% (*n* = 4)	20.4% (*n* = 21)	−13.4%	4.976 _(1)_	*p* = 0.026	0.18
The fact that the President of the Republic or the Prime Minister promoted the vaccine	10.5% (*n* = 6)	5.8% (*n* = 6)	4.7%	1.169 _(1)_	*p* = 0.280	
The fact that the vaccination was promoted in my social media network	5.3% (*n* = 3)	3.9% (*n* = 4)	1.4%	0.167 _(1)_	*p* = 0.683	
The fact that a trusted news source promoted the vaccine	7% (*n* = 4)	4.9% (*n* = 5)	2.1%	0.323 _(1)_	*p* = 0.570	

Δ%: Percentage difference between physicians and other HCPs.

**Table 3:** Flu vaccination acceptance and motivations behind COVID-19 vaccination behaviors (*n* = 160).

Motivation	(*n*)% Vaccinated against Flu in the Past Yes/Definitely Yes	(*n*)% NOT Vaccinated against Flu in the Past Yes/Definitely Yes	Δ%	χ^2^_(d.f.)_	*p*	Cramer’s V
Being convinced that getting vaccinated helped protect vulnerable members of my community	97.6% (*n* = 80)	97.4% (*n* = 76)	0.2%	0.003 _(1)_	*p* = 0.960	
Being convinced that the vaccine will serve to protect my health	97.6% (*n* = 80)	89.7% (*n* = 70)	7.9%	4.169 _(1)_	*p* = 0.041	0.16
Thinking that the health authorities were trustworthy on this argument	65.9% (*n* = 54)	51.3% (*n* = 40)	14.6%	3.502 _(1)_	*p* = 0.061	
Being convinced that the vaccine had been rigorously tested	56.1% (*n* = 46)	51.3% (*n* = 40)	4.8%	1.671 _(1)_	*p* = 0.541	
The fact that a trusted health care worker suggested I get vaccinated	24.4% (*n* = 20)	32.1% (*n* = 25)	7.7%	1.161 _(1)_	*p* = 0.081	
The fact that someone I knew got sick with COVID-19	22% (*n* = 18)	25.6% (*n* = 20)	3.6%	0.301 _(1)_	*p* = 0.584	
The fact that someone I knew was hospitalized due to COVID-19	20.7% (*n* = 17)	23.1% (*n* = 18)	2.4%	0.129 _(1)_	*p* = 0.720	
The fact that someone I knew died due to COVID-19	14.6% (*n* = 12)	16.7% (*n* = 13)	2.1%	0.125 _(1)_	*p* = 0.723	
The fact that the President of the Republic or the Prime Minister promoted the vaccine	4.9% (*n* = 4)	10.3% (*n* = 8)	5.4%	1.667 _(1)_	*p* = 0.197	
The fact that the vaccination was promoted in my social media network	3.7% (*n* = 3)	5.1% (*n* = 4)	1.4%	0.206 _(1)_	*p* = 0.650	
The fact that a trusted news source promoted the vaccine	4.9% (*n* = 4)	6.4% (*n* = 5)	1.5%	0.177 _(1)_	*p* = 0.674	

Δ%: Percentage difference between vaccinated and not vaccinated in the past.

## Data Availability

Not applicable.

## Supplementary Materials

The following supporting information can be downloaded at: https://www.mdpi.com/article/10.3390/vaccines10030467/s1, File S1: Questionnaire.
