# Supplementary material for: Exploring the Motivational Roots of Getting Vaccinated against COVID-19 in a Population of Vaccinated Pediatric Healthcare Professionals: Evidence from an Italian Cross-Sectional Study
Source: Vaccines (Basel). 2022 Mar 18;10(3):467. doi: 10.3390/vaccines10030467 (PMC8952861; doi:10.3390/vaccines10030467)
Supplement: Supplementary file 1 [file vaccines-10-00467-s001.zip › vaccines-1612804-supplementary.pdf]

Supplementary Material

1. Questionnaire (English version)

To start, we ask you some questions, in order to have a socio-demographic and professional profile

1.Gender:

- ☐ Male
- ☐ Female

2.Age:_______

3.Occupation:

- ☐ Physician
- ☐ Nurse
- ☐ Obstetrician
- ☐ Allied healthcare workers
- ☐ Other: _____

In this section, we present you some questions about the COVID-19 vaccine

4.If you think to the COVID-19 vaccine… (select one option):

- ☐ a. I have already been vaccinated, first and second dose
- ☐ b. I have already been vaccinated, only the first dose
- ☐ c. I haven't been vaccinated yet, but I have already adhered to the vaccination campaign
- ☐ d. I haven't adhered to the vaccination campaign, but I will in the future
- ☐ e. I haven't decided on this yet
- ☐ f. I haven't adhered to the vaccination campaign, and I will not join in the future

(Filter: only if the respondent in question 4. have chosen a. or b. or c.)

5.Below there are some possible reasons that could motivated people to get vaccinated against COVID-19. For each of the following reasons, indicate how much these incentives influenced your decision to get vaccinated against COVID-19, on a scale of 1 to 5 (from 1 -definitely not- to 5 -definitely yes)

**Items**	**Definitely not**				**Definitely yes**
1. Be convinced that the vaccine had been rigorously tested	1	2	3	4	5
2. To think that the health autorities were trustworthy on this argument	1	2	3	4	5

3. Be convinced that getting vaccinated helped protect vulnerable members of my community	1	2	3	4	5
4. The fact that a trusted health care worker suggest me to get vaccinated	1	2	3	4	5
5. The fact that someone I knew died due to COVID-19	1	2	3	4	5
6. The fact that someone I knew got sick with COVID-19	1	2	3	4	5
7. The fact that someone I knew was hospitalized due to COVID-19	1	2	3	4	5
8. The fact that a trusted news source promoted the vaccine	1	2	3	4	5
9. The fact that the President of the Republic or the Prime Minister promoted the vaccine	1	2	3	4	5
10. The fact that the vaccination was promoted in my social media network	1	2	3	4	5
11. Be convinced that the vaccine will serve to protect my health	1	2	3	4	5

Thinking about when you did the anti-COVID-19 vaccine, state how much you felt the following emotional states using a scale from 0 (not at all) to 100 (a lot)

	Not at all				A lot
	0	25	50	75	100
Happy					
Proud					
Grateful					
Worried					
Satisfied					
Safe					
Forced					
Exited					
Calm					
Impatient					

2. Questionnaire (Italian version)

Per iniziare, le chiediamo di fornirci alcuni dati su di lei, al fine di avere un profilo socio-demografico e professionale:

Genere:

- ○ Uomo
- ○ Donna

Età in anni compiuti (in cifre):

____

Professione:

- ○ Medico
- ○ Infermiere/a
- ○ Ostetrico/a
- ○ OSS
- ○ Altro ____

Pensando al vaccino contro il COVID-19 (selezioni una delle seguenti risposte):

- ◯ Ho già fatto il vaccino e il richiamo
- ◯ Ho già fatto il vaccino (solo la prima dose)
- ◯ Non sono ancora stato vaccinato, ma ho già aderito al piano vaccinale
- ◯ Non ho ancora aderito al piano vaccinale, ma sicuramente lo farò in futuro
- ◯ Non ho ancora deciso in merito
- ◯ Non ho ancora aderito al piano vaccinale, né lo farò in futuro

Visualizza questa domanda:

If Pensando al vaccino contro il COVID-19 (selezioni una delle seguenti risposte): = Non ho ancora aderito al piano vaccinale, ma sicuramente lo farò in futuro

Or Pensando al vaccino contro il COVID-19 (selezioni una delle seguenti risposte): = Non ho ancora deciso in merito

Or Pensando al vaccino contro il COVID-19 (selezioni una delle seguenti risposte): = Non ho ancora aderito al piano vaccinale, né lo farò in futuro

Di seguito trova una serie di possibili motivazioni che potrebbero essere determinanti nello spingere le persone che hanno dubbi riguardo al vaccino anti-COVID-19 a vaccinarsi. Per ognuna delle seguenti motivazioni, indichi quanto ritiene che ognuno di questi incentivi potrebbe aumentare la probabilità che lei

decida di vaccinarsi contro il COVID-19, su una scala da 1 a 5 (dove 1=decisamente no; 5= decisamente si). ***Mi motiverebbe a fare il vaccino...***

	1 Decisamente No	2	3	4	5 Decisamente sì
... se fossi convinto che il vaccino sia stato rigorosamente testato	○	○	○	○	○
... se pensassi che le autorità sanitarie siano affidabili	○	○	○	○	○
... se fossi convinto che farsi vaccinare aiutasse a proteggere i membri vulnerabili della mia comunità	○	○	○	○	○
... se un operatore sanitario di fiducia mi dicesse di vaccinarmi	○	○	○	○	○
... se un mio conoscente fosse morto a causa di COVID-19	○	○	○	○	○
... se un mio conoscente fosse ricoverato in ospedale a causa di COVID-19	○	○	○	○	○
... se una testata giornalistica di cui mi fido promuovesse la vaccinazione	○	○	○	○	○
... se il Presidente della Repubblica o il Presidente del Consiglio promuovessero il vaccino	○	○	○	○	○
... se la vaccinazione venisse promossa nella mia rete di amici dei social network	○	○	○	○	○

... se fossi convinto che il vaccino servisse a tutelare la mia salute	○	○	○	○	○

Interruzione di pagina

Visualizza questa domanda:

If Pensando al vaccino contro il COVID-19 (selezioni una delle seguenti risposte): = Ho già fatto il vaccino e il richiamo

Or Pensando al vaccino contro il COVID-19 (selezioni una delle seguenti risposte): = Ho già fatto il vaccino (solo la prima dose)

Pensando a quando si è sottoposto al vaccino anti-Covid19, indichi quanto si è sentito nei seguenti stati usando la scala da 0 (per nulla) a 100 (molto):

	Per nulla				Molto
	0	25	50	75	100
Felice					
Orgoglioso					
Grato					
Preoccupato					
Soddisfatto					
Al sicuro					
Forzato					
Emozionato					
Tranquillo					
Impaziente					
